# Assessment of Colistin Heteroresistance among Multidrug-Resistant *Klebsiella pneumoniae* Isolated from Intensive Care Patients in Europe

**DOI:** 10.3390/antibiotics13030281

**Published:** 2024-03-20

**Authors:** Anouk J. M. M. Braspenning, Sahaya Glingston Rajakani, Adwoa Sey, Mariem El Bounja, Christine Lammens, Youri Glupczynski, Surbhi Malhotra-Kumar

**Affiliations:** Laboratory of Medical Microbiology, Vaccine & Infectious Disease Institute, Universiteit Antwerpen, 2610 Antwerp, Belgium

**Keywords:** colistin resistance, mechanisms, mgrB, population analysis profiling, whole-genome sequencing

## Abstract

Heteroresistance (HR) to colistin is especially concerning in settings where multi-drug-resistant (MDR) *K. pneumoniae* are prevalent and empiric use of colistin might lead to treatment failures. This study aimed to assess the frequency of occurrence of colistin HR (CHR) among (MDR) *K. pneumoniae* (*n* = 676) isolated from patients hospitalized in 13 intensive care units (ICUs) in six European countries in a clinical trial assessing the impact of decolonization strategies. All isolates were whole-genome-sequenced and studied for in vitro colistin susceptibility. The majority were colistin-susceptible (CS) (*n* = 597, MIC ≤ 2 µg/mL), and 79 were fully colistin-resistant (CR) (MIC > 2 µg/mL). A total of 288 CS isolates were randomly selected for population analysis profiling (PAP) to assess CHR prevalence. CHR was detected in 108/288 CS *K. pneumoniae*. No significant association was found between the occurrence of CHR and country, MIC-value, K-antigen type, and O-antigen type. Overall, 92% (617/671) of the *K. pneumoniae* were MDR with high prevalence among CS (91%, 539/592) and CR (98.7%, 78/79) isolates. In contrast, the proportion of carbapenemase-producing *K. pneumoniae* (CP-Kpn) was higher among CR (72.2%, 57/79) than CS isolates (29.3%, 174/594). The proportions of MDR and CP-Kpn were similar among CHR (MDR: 85%, 91/107; CP-Kpn: 29.9%, 32/107) and selected CS isolates (MDR: 84.7%, 244/288; CP-Kpn: 28.1%, 80/285). WGS analysis of PAP isolates showed diverse insertion elements in *mgrB* or even among technical replicates underscoring the stochasticity of the CHR phenotype. CHR isolates showed high sequence type (ST) diversity (Simpson’s diversity index, SDI: 0.97, in 52 of the 85 STs tested). CR (SDI: 0.85) isolates were highly associated with specific STs (ST101, ST147, ST258/ST512, *p* ≤ 0.003). The widespread nature of CHR among MDR *K. pneumoniae* in our study urge the development of rapid HR detection methods to inform on the need for combination regimens.

## 1. Introduction

*Klebsiella pneumoniae* frequently cause community-acquired and nosocomial infections such as pneumonia, urinary tract infection, liver abscesses and bloodstream infections [1]. In recent years, antimicrobial resistance in *K. pneumoniae* has become problematic [2,3]. In particular, resistance to carbapenems is frequently associated with resistance to multiple classes of other antibiotics which leads to limited possibilities for clinical management as alternative treatment options are limited and lead to higher rates of treatment failures [4]. In such cases, last-resort antibiotics such as colistin [5,6] may be used in association with other antibiotics. However, the high worldwide prevalence of carbapenem-resistant, multi-drug resistant (MDR) *K. pneumoniae* has fueled and increased the use of colistin over the last years, accelerating the emergence of isolates resistant to this compound [3]. Besides full colistin resistance (CR), colistin heteroresistance (CHR) has also been increasingly reported over the last years [7,8]. Heteroresistance (HR) is defined as a phenotype in which a bacterial isolate contains subpopulations of cells that show a substantial reduction in antibiotic susceptibility compared with the main population (minimum inhibitory concentration (MIC) increase of at least eight-fold), allowing for these cells to grow in the presence of the antibiotic [9]. Detection of these subpopulations can be challenging and raise concern as their frequency may rise during antibiotic exposure and possibly lead to treatment failure [9,10,11]. However, the relevance of CHR in causing reduced clinical effectiveness and negatively affecting the treatment outcome remains controversial [7]. Recent studies have highlighted the high prevalence of CHR, but these were often limited by small sample size (mostly relying on local data) and limited investigation of the characteristics of the CHR *K. pneumoniae* strains. In this study, we took advantage of the unique availability of a large collection of clinical isolates from various intensive care units (ICUs) across Europe to gain more insight into the frequency of occurrence of CHR among colistin-sensitive (CS) *K. pneumoniae* and to investigate its possible association with epidemiological and clinical characteristics such as country of origin, the different therapeutic intervention strategies to which the patients were exposed during their stay in the ICU, and sequence type (ST). We also investigated CHR *K. pneumoniae* strains for their possible association with capsular polysaccharide antigen types (K-antigen type), lipopolysaccharide antigen types (O-antigen type), and colistin MIC value.

## 2. Materials and Methods

### 2.1. Sample and Isolate Collection

*Klebsiella pneumoniae* (*n* = 676) collected during a clinical trial as part of the R-GNOSIS project (Resistance in Gram-Negative Organisms: Studying Intervention Strategies) (NCT02208154) were utilized. Isolates were collected from patients hospitalized in ICUs from 13 sites in six European countries: Belgium (*n* = 239), Spain (*n* = 201), Portugal (*n* = 61), Italy (*n* = 143), Slovenia (*n* = 9), and United Kingdom (*n* = 23) between 1 December 2013 and 31 May 2017. The reason for the lower number of isolates in Slovenia and United Kingdom were two-fold. Firstly, only a single ICU in each country participated in the trial in contrast to other included countries. Secondly, the baseline colonization rates with *Enterobacteriaceae* resistant to third-generation cephalosporins in Slovenia and the United Kingdom were rather low [12]. ICU patients with an expected duration of invasive mechanical ventilation of minimally 24 h were included while those who were not intubated nor mechanically ventilated and those that stayed in the unit for less than 24 h were not included in the study. In the primary R-GNOSIS clinical trial, participants were assigned to three different groups of intervention, namely chlorhexidine mouthwash (CHX), selective oropharyngeal decontamination (SOD), and selective digestive tract decontamination (SDD), aimed at reducing the risk of bloodstream infections due to MDR-Gram-negative bacteria among ventilated patients in ICUs with moderate to high prevalence of antibiotic resistance [12]. Both SDD and SOD topical decontamination treatments consist of an oropharyngeal paste and enteral suspension containing antimicrobials that includes colistin (as well as tobramycin and nystatin) (Supplementary Table S1). After a baseline period (6–14 months), each intervention was implemented for periods of six months in a random order for each ICU and was separated by a one-month wash-out/in period. Samples and subsequently culture-isolated microorganisms were categorized into three categories: surveillance, point prevalence survey (PPS), or clinical. Surveillance samples were from patients directly undergoing interventions (i.e., who had received the decontamination treatment) and were taken twice weekly. PPS samples were collected monthly from all patients present in the ward at that time, including those not undergoing the interventions. Clinical samples were those obtained when needed for the clinical management of the patients [12]. Isolates originated from various body sites: respiratory (more specifically aspirate, throat swab, sputum, bronchoalveolar lavage or non-directed bronchoalveolar lavage), blood, groin, or rectum.

### 2.2. MIC Determination

All 676 first patient isolates were subjected to colistin susceptibility testing using either an E-test (bioMérieux, Marcy l’Etoile, France) or automatic testing method (BD Phoenix^TM^ (BD Diagnostics, Le Pont de Claix, France), Sensititre^TM^ (Thermo Fisher Scientific, Waltham, MA, USA), Vitek^®^ (bioMérieux, Marcy l’Etoile, France), MicroScan (Beckman Coulter, San Diego, CA, USA)) [12]. Of the 676 isolates, 79 (11.7%) were determined to be fully CR. It must be noted, however, that these methods are not recommended for colistin susceptibility testing and it has been shown that they may underestimate CR rates [13], with the exception of Sensititre^TM^ which has been found to perform well when compared to the reference method [14]. Therefore, to confirm the susceptibility of the selected isolates (see population analysis profiling assay), the colistin MIC was also determined by broth microdilution using the MICRONAUT MIC-Strip Colistin (MERLIN Diagnostika GmbH, Berlin, Germany). In the case of one skipped well, the result was determined disregarding this well (i.e., the skipped well was not seen as the lowest concentration showing no growth). In the case of multiple skipped wells, the test was repeated. An isolate was classified as susceptible (MIC ≤ 2 mg/L) or resistant (MIC > 2 mg/L) based on the epidemiological cut-off (ECOFF) values provided by EUCAST [15]. Two CS strains, *Escherichia coli* ATCC 25922 (MIC: 0.25–2 mg/L) and *P. aeruginosa* ATCC 27853 (MIC: 0.5–4 mg/L), and two CR strains, *K. pneumoniae* 08400 (MIC: 64 mg/L) and *E. coli* NCTC 13846 *mcr-1* positive (MIC: 4 mg/L), were used as controls [16].

### 2.3. Population Analysis Profiling (PAP) Assay

For HR screening, we randomly selected 288 isolates of the 597 CS (MIC ≤ 2 mg/L) isolates for population analysis profiling (PAP) based on the two following criteria: (1) Number of isolates selected per country had to reflect the proportion of isolates contributed by each country and also (2) to match with the distribution of isolates found during the baseline and the three intervention strategies (Supplementary Table S1).

For the PAP assay, 0.5 MacFarland (McF) bacterial suspensions were prepared using the BD PhoenixSpec™ nephelometer (BD Diagnostics, Le Pont de Claix, France) starting from an overnight culture on Columbia blood agar (Oxoid Ltd., Basingstoke, UK) with 5% defibrinated horse blood (International Medical Products, Oudergem, Belgium). A 100 µL aliquot of this suspension was spirally plated using the Eddy Jet (IUL instruments S.A., Barcelona, Spain) on a series of cation adjusted Mueller Hinton Agar (CAMHA) (BD Diagnostics, Le Pont de Claix, France) plates containing colistin (Sigma-Aldrich, St. Louis, MO, USA) in increasing concentrations (0 mg/L, colistin free; 1 mg/L; 2 mg/L; 4 mg/L, 8 mg/L; 16 mg/L). The number of colonies were counted after 24 h of aerobic incubation of the plates at 37 °C, and a graph of the log_10_ CFU/mL was plotted against the increasing colistin concentrations. One CS strain, *E. coli* ATCC 25922 (MIC: 0.25–2 mg/L), one CR strain, *K. pneumoniae* 08400 (MIC: 64 mg/L), and two CHR strains, *K. pneumoniae* ATCC 13883 (MIC: 1 mg/L) and *K. pneumoniae* IT0244CP (MIC: 0.5 mg/L), were used as quality controls [16].

**Figure 1:**
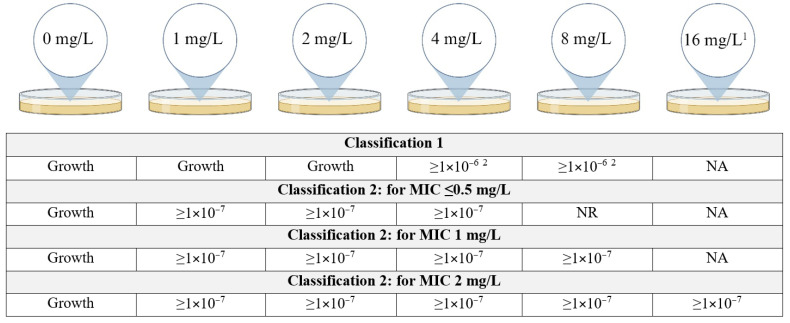
Classification schemes for colistin heteroresistance (CHR). Figure describes the growth requirements, more specifically the frequency of growth required, for an isolate to be determined CHR. In the case there is no frequency requirement at a specific concentration of colistin but only the requirement that there is visible growth on the plate, this is indicated as “Growth”. For Classification 2, the requirements to be fulfilled depend on the MIC for colistin of the isolates. NR = no requirement, NA = not applicable, MIC = minimum inhibitory concentration. ^1^ Plates containing 16 mg/L of colistin were only included for isolates with a MIC of 2 mg/L. ^2^ Frequency of ≥1 × 10^−6^ only required for either 4 mg/L or 8 mg/L, not for both though it is allowed. Created with BioRender.com.

Since there is no consensus on the definition of CHR, we used two previously defined schemes (Figure 1). Classification 1 (C1) was based on the classification as used by Band et al. [17] while Classification 2 (C2) was based on Andersson et al. [9] with the additional requirement that growth at a frequency of minimally 1 × 10^−7^ must be observed at least at 4 mg/L even if the eight-fold MIC of the isolate was below 4 mg/L. More specifically, this means that for a MIC of ≤0.5 mg/L, growth at a frequency of 1 × 10^−7^ must be observed on plates containing 1, 2 and 4 mg/L of colistin, for a MIC of 1 mg/L the former must be observed additionally on the plates containing 8 mg/L of colistin and for a MIC of 2 mg/L also on plates containing 16 mg/L. This additional requirement was put in place to account for the possibility of false positives occurring due to the inoculum effect. The frequency was determined for each concentration of colistin using the following calculation:

$$Frequency=\frac{\mathrm{CFU}/\mathrm{mL}\;colistin\;plate}{\mathrm{CFU}/\mathrm{mL}\;colistin\;free\;plate}$$

### 2.4. Whole-Genome Sequencing

Whole-genome sequencing (WGS) was employed to determine the ST, O-antigen type, and K-antigen type of the 676 *K. pneumoniae* isolates as well as to look for mutations in known (hetero)resistance genes for colistin. Strains were cultured on CAMHA and incubated for 16–20 h at 35–37 °C. After incubation, one colony of the CAMHA plate was inoculated in a polypropylene tube containing 4 mL of cation adjusted Mueller Hinton Broth (CAMHB) (BD Diagnostics, Le Pont de Claix, France) and incubated again for 16–20 h at 35–37 °C. A negative growth control was prepared containing only 4 mL of CAMHB. DNA extraction was performed using the MasterPure^TM^ Complete DNA and RNA Purification Kit (Epicentre Biotechnologies, Madison, WI, USA) following manufacturer’s instructions. DNA was further purified using the DNA Clean & ConcentratorTM-10 kit (Zymo Research, Irvine, CA, USA) following instructions as provided by the manufacturer. Library preparation was performed using the Nextera^®^ XT DNA Sample Preparation Kit and the Nextera^®^ XT Index Kit v2 Set A (Illumina, San Diego, CA, USA) in conjunction with the Zephyr^®^ G3 NGS liquid handler (PerkinElmer, Waltham, MA, USA), containing heating and shaking modules controlled by the Inheco Multi TEC Controller (INHECO GmbH, Martinsried, Germany). Sequencing was performed with the MiSeq sequencer (Illumina, San Diego, CA, USA). Data were analyzed using BacPipe v6.0 [18] and CLC Genomics Workbench software (Qiagen, Hilden, Germany).

### 2.5. Statistical Analysis

To determine whether there was an association between CHR/CR and country, MIC-value, intervention strategy, ST, O-antigen, or K-antigen type and between country, ST, and MIC-value, a Pearson Chi-square test or Fisher Exact test were used. When conditions for a Chi-square test or Fisher Exact test were not met (i.e., no cells with expected values < 1, and no more than 20% of cells with values < 5), a Monte Carlo simulation was used. Since this is not an exact method, in contrast to the regular Fisher Exact test, the *p*-value was given with the 99% confidence interval (99% CI). *p*-values less than 0.05 were considered statistically significant. In the case of a statistically significant association, a pairwise z-test with Bonferroni correction was used to assess which groups had an association. All analyses were performed using the IBM^®^ SPSS^®^ Statistics software version 28.0.1.1 (IBM Corp., Armonk, NY, USA).

## 3. Results

### 3.1. Population Analysis Profiling (PAP)

**Figure 2:**
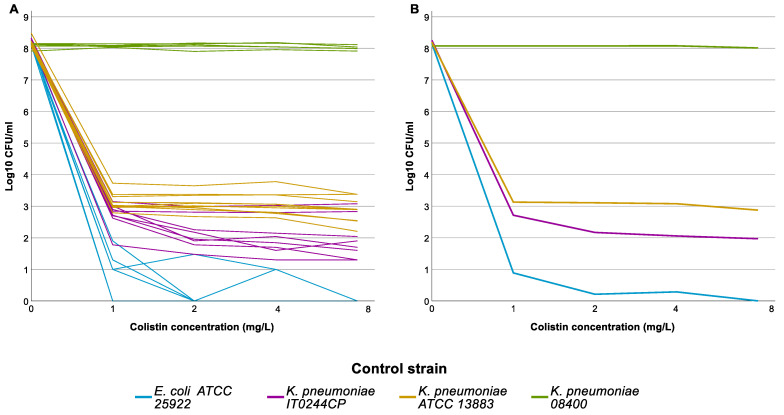
Population analysis profiling (PAP) assay results of the control strains. Graph represents the log_10_ colony forming units (CFU)/mL per concentration of colistin used in the agarplates of the PAP assay. (**A**) Individual results for each strain for each run, graph illustrates the intra-run variation for the different control strains. (**B**) Average result for each control strain, graph illustrates the overall result of the control strains.

The 288 selected isolates were tested by PAP assay in 11 distinct runs. Results of the PAP assay for the control strains in the 11 full runs are shown in Figure 2. One of the two HR control strains, IT0244CP, grew on all colistin-containing plates in all 11 runs, though the frequency varied from run to run. The frequency threshold of 1 × 10^−6^ was only reached twice (C1); still, IT0244CP always fulfilled C2. The second CHR control strain *K. pneumoniae* ATCC 13883 also showed some variability in the frequency of the resistant subpopulation but fulfilled C1 in all 11 runs.

Overall, out of the 288 isolates tested, 25 were classified as being CHR based on the more stringent criteria of C1 whilst 108 isolates were classified as being CHR based on the less stringent C2 criteria. All isolates that fulfilled C1 also fulfilled C2 (Table 1 and Supplementary Table S2).

### 3.2. High Prevalence of Multi-Drug-Resistant and Carbapenemase-Producing Isolates

Overall, 617 out of 671 (92%) of the *K. pneumoniae* isolates, for which the information was available, were classified as MDR based on Magiorakos et al. [19]. MDR prevalence was high among both CS (91%, 539/592) and CR (98.7%, 78/79) isolates. In contrast, the proportion of carbapenemase-producing *K. pneumoniae* (CP-Kpn) was higher among CR isolates (72.2%, 57/79) than among CS isolates (29.3%, 174/594). On the other hand, similar proportions of MDR and of CP-Kpn were observed among CHR (MDR: 85%, 91/107; CP-Kpn: 29.9%, 32/107) and selected CS isolates (MDR: 84.7%, 244/288; CP-Kpn: 28.1%, 80/285) (Supplementary Tables S3 and S4).

### 3.3. Analysis of Association between Colistin-Resistant K. pneumoniae, ST, O-Antigen Type and K-Antigen Type

**Figure 3:**
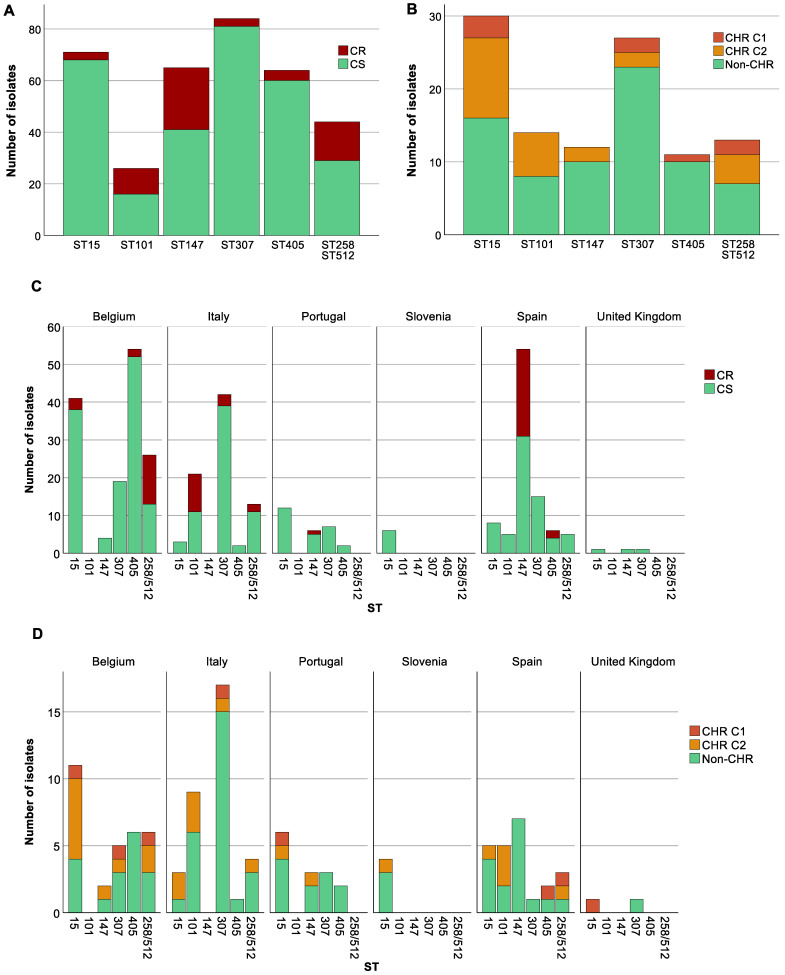
Distribution of isolates across sequence types (STs). Graphs show the number of isolates per ST as well as the number of colistin-resistant (CR)/colistin-heteroresistant (CHR) and colistin-susceptible (CS)/non-CHR isolates. Only STs with a statistically significant association with CR and/or CHR are shown. Of note, Classification 1 (C1) + Classification 2 (C2) represents the total amount of isolates fulfilling C2 whilst C2 alone represents isolates only fulfilling C2. (**A**) CR per ST, (**B**) CHR per ST, (**C**) CR per ST per country, Slovenia (*n* = 9) and United Kingdom (*n* = 23), were not taken into further consideration due to the low number of isolates, (**D**) CHR per ST per country.

To investigate the distribution of CR within various STs, we studied those STs that were represented at least 10 times in our collection. The 79 CR *K. pneumoniae* isolates in our study were distributed among 14 different STs with three STs (ST101, ST147, ST258/512) accounting for 62% (*n* = 49) of the total CR isolates. CR was significantly associated with specific STs (*p* at least 0.003); for instance, ST101 (CR: 12.7%, 10/79, CS: 2.7%, 16/597), ST147 (CR: 30.4%, 24/79, CS: 6.9%, 41/597) and ST258/ST512 (CR: 19%, 15/79, CS: 4.9%, 29/597) (*p* for all <0.05) showed higher proportions among CR strains than among CS strains. Conversely, ST15 (CR: 3.8%, 3/79, CS: 11.4%, 68/597), ST307 (CR: 3.8%, 3/79, CS: 13.6%, 81/597) and ST405 (CR: 5.1%, 4/79, CS: 10.1%, 60/597) (*p* for all <0.05) had higher proportions of CS isolates compared to CR. We could not document exclusive association either with CS or with CR of any ST that was present in the collection at least 10 times (Figure 3A).

CS isolates (*n* = 597) were distributed over 87 STs (Simpson’s diversity index, SDI: 0.94) while the 288 CS isolates tested on the PAP assay belonged to 85 STs (SDI: 0.97). Of these, CHR was found among 52 different STs and showed a remarkably higher genetic diversity (SDI: 0.97) compared to ST distribution among CR isolates (SDI: 0.85). Ten STs (ST11, ST15, ST45, ST101, ST147, ST258/512, ST307, ST405, ST409 and ST437) were common to both CR and CHR isolates.

Besides an association between CR and STs, country and STs also showed a strong association (*p* at least <0.001). *K. pneumoniae* belonging to ST15 were spread across all six countries; however, the proportion of ST15 was higher in Belgium (17.2%, 41/239) and Portugal (19.7%, 12/61) than in Italy (2.1%, 3/143) and Spain (4%, 8/201) (*p* < 0.05). ST307 was present in five of the six countries. In this case, the proportion of ST307 isolates in Italy (29.4%, 42/143) was significantly different compared to Belgium (7.9%, 19/239) and Spain (7.5%, 15/201) (*p* < 0.05) but not when compared to Portugal (11.5%, 7/61) (*p* > 0.05). ST147 was not found in Italy and most of the isolates were from Spain (26.9%, 54/201) for which the proportion also significantly differed from both Belgium (1.7%, 4/239) and Portugal (9.8%, 6/61) (*p* < 0.05). In contrast to the previous STs which were found in almost all countries, ST101 and ST258/ST512 were only found in two and three countries, respectively. ST101 showed a statistically significant difference between the two countries with a higher proportion in Italy (14.7%, 21/143) compared to Spain (2.5%, 5/201) (*p* < 0.05). ST258/ST512 showed a statistically significant difference between Belgium (10.5%, 25/239) and Spain (2.5%, 5/201) (*p* < 0.05), but not between Italy (9.1%, 13/143) and the other two countries (*p* > 0.05). Among ST101, all CR isolates were isolated in Italy, for ST147 all but one were isolated in Spain (one CR isolate from Portugal), and for ST258/ST512 all but two from Belgium (two CR isolates from Italy) (Figure 3C).

For the K-antigen and O-antigen types, a tight association is known to exist with specific STs and therefore the significant differences found in the proportion of CR and CS within a ST were likewise reflected in similar differences in the proportion of K and O antigen types (*p* for both ≤ 0.001).

### 3.4. A Higher Proportion of CR among Specific STs Was Not Reflected in CHR Proportions

For CHR and ST, an association was found (*p* at least 0.021). In contrast, CHR isolates did not show any clear association with K-antigen and O-antigen types (*p* ≥ 0.169). ST307 and ST405 were found to have a higher proportion of non-CHR isolates (ST307: CHR: 3.7%, 4/108, non-CHR: 12.8%, 23/180, *p* < 0.05 and ST405: CHR: 0.9%, 1/108, non-CHR: 5.6%, 10/180, *p* < 0.05) (Figure 3B). Selected isolates from both STs were spread across different countries (Figure 3D). There was no association between STs found to have a higher proportion of CR isolates and CHR. Of the 39 isolates tested for CHR with STs ST101, ST147, and ST258/ST512, only 2 were determined to be CHR based on C1 and 14 exclusively based on C2.

### 3.5. Analysis of Association between Colistin (Hetero)Resistant K. pneumoniae, Country, Intervention Strategy, and MIC-Value

**Figure 4:**
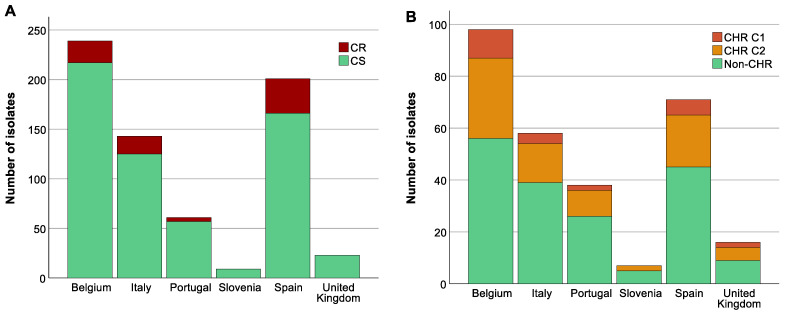
Distribution of isolates across countries. Graphs show the number of isolates per country as well as the number of colistin-resistant (CR)/colistin-heteroresistant (CHR) and colistin-susceptible (CS)/non-CHR isolates. Of note, Classification 1 (C1) + Classification 2 (C2) represents the total amount of isolates fulfilling C2 whilst C2 alone represents isolates only fulfilling C2. (**A**) CR per country, (**B**) CHR per country.

The highest proportion of CR *K. pneumoniae* was found in Spain (44.3%, 35/79, *p* < 0.05) (Figure 4A). There was also a difference in the distribution of CR across the baseline, CHX, SDD, and SOD (*p* = 0.001) with a higher proportion of CR isolates in patients in all three intervention strategy groups compared to the baseline period (*p* < 0.05) but no significant difference in distribution of CR was identified between the three intervention groups (Supplementary Figure S1). This could mainly be explained by the increase in the proportion of CR ST147 isolates in Spain during the intervention periods (for all three groups) compared to the baseline (8.8–15.6% versus 3.8%). In contrast, no significant association was found between increase in CHR with any country (*p* > 0.723) (Figure 4B) or between CHR and any intervention strategy (*p* > 0.668). CHR isolates were present in the highest proportions in ST15 followed by ST101 (Figure 3B). Of note, CHR isolates that matched the stricter definition (i.e., C1) were found in higher proportion in ST15 followed by ST307, but they were not found at all among the ST101 and ST147 isolates (Figure 3B).

**Figure 5:**
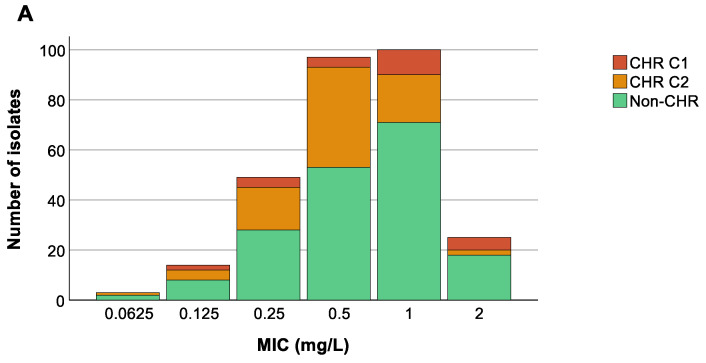

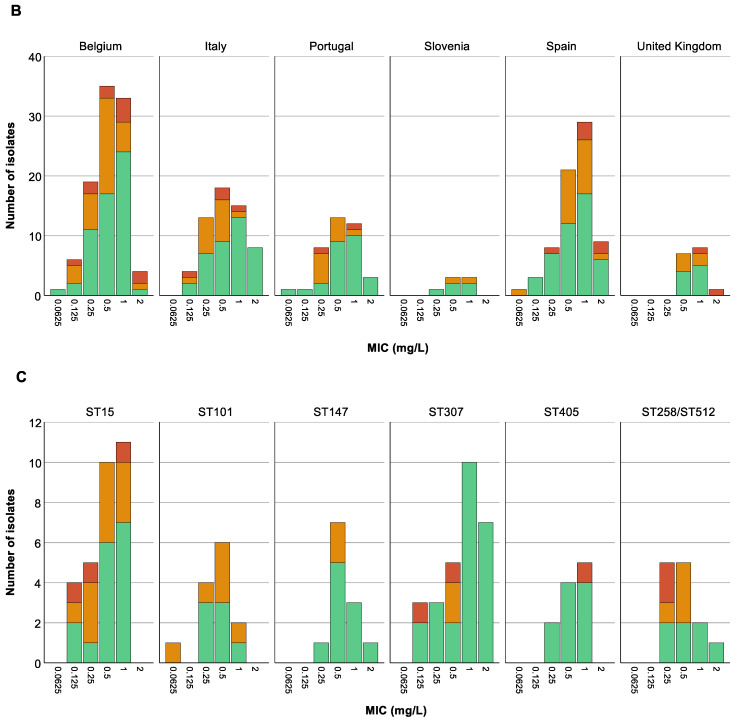
Distribution of isolates across minimum inhibitory concentration (MIC) values. Graphs show the number of isolates per MIC as well as the number of colistin-heteroresistant (CHR) and non-CHR isolates. Of note, Classification 1 (C1) + Classification 2 (C2) represents the total amount of isolates fulfilling C2 whilst C2 alone represents isolates only fulfilling C2. (**A**) CHR per MIC value, (**B**) CHR per MIC value per country, (**C**) CHR per MIC value per sequence type (ST). Only STs with a statistically significant association with CR and/or CHR are shown.

We could not find any association between the colistin MIC value of the 288 selected isolates and countries (*p* at least 0.599) (Figure 5B), nor was an association present between ST and MIC values (*p* at least 0.08) (Figure 5C). Remarkably, however, there was a trend towards higher colistin MIC values for isolates classified as CHR based on C1 compared to CHR isolates fulfilling only C2 (Figure 5A).

### 3.6. Colistin Resistance Mechanisms in Resistant Subpopulations

Isolates AN1505CP2 and IT0244CP were previously already determined to be CHR [16]. In this study, these findings were confirmed with both isolates fulfilling C1, and colonies from each plate were sequenced (data not published). Mutations found in colistin resistance-associated genes (*mgrB*, *phoP*, *phoQ*, *pmrA*, *pmrB*, *pmrD*, *arnA*, *kpnEF*, *kpnF*, *crrB* and *acrB*) are summarized in Table 2. The resistant subpopulations of AN1505CP2 and IT0244CP both showed disruptions of the *mgrB* gene by various insertion sequences (IS1R, IS1X2, ISKpn34, IS903B).

For IT0244CP, the disruption of *mgrB* was found across different concentrations of colistin in the same PAP assay (but these were linked with different insertion sequences and interruption at different nucleotide positions) (Table 2). In contrast, the resistant subpopulation of AN1505CP2 did show differences in the type of mutations found in *mgrB* between different concentrations of colistin in the same PAP assay run. To assess whether the mechanism of resistance was ST-specific or stochastic, we looked into CR isolates with the same ST as IT0244CP (ST409). For AN1505CP2 (ST323), in our collection, we could not find any CR isolates that belong to the same ST (Table 2). For ST409, both the CR and CHR isolates showed disruptions of *mgrB* by insertion sequences. However, we did not find reproducibility in the mutations selected even for the same (CHR) isolate between technical replicates of the PAP assay, highlighting the stochasticity of the selection process.

## 4. Discussion

In general, research around HR has been proven to be challenging, especially due to a lack of standardization of identification methods as well as of precise classification criteria. In the past, it has therefore also been difficult to acquire reliable estimates on the prevalence of HR as the sample sizes of the performed studies have been generally small and, due to a lack of standardization, both in definitions of HR but also in assays, it has been difficult to compile and compare different studies [1,9]. There are various ways through which HR can be screened, including methods that are used routinely for example for MIC determination, but they have been deemed to be not performant enough [9,20,21].

A strength of this study was its large sample size compared to previous studies on CHR using the PAP assay [22,23,24,25]. Within this study, two different classification schemes for CHR were used. C1 was considered to be more stringent due to the higher frequency requirement, and the number of isolates fulfilling this more stringent classification CHR was thus limited (*n* = 25). The majority of isolates categorized as CHR fulfilled the second less stringent classification (*n* = 108) which was considered to be less stringent due to a lower frequency requirement though this classification included an additional requirement with regard to the concentration at which growth should occur. It has to be acknowledged that there were also numerous isolates that could not be classified as CHR by any of these two classifications but did show growth on PAP assay plates > 2 mg/L colistin. Most often, this was growth at frequency < 1 × 10^−7^ (often only one colony). We cannot rule out that these colonies were spontaneous mutants and were thus not caused by the isolate being CHR or were due to an inoculum effect. They were therefore not classified as CHR.

This study was also unique in the investigated associations, which, to our knowledge, have not been studied previously in *K. pneumoniae.* A study on CHR in *A. baumannii* reported the absence of association between CHR and MIC value and clonal complexes [24]; however, these results are not necessarily applicable to CHR in *K. pneumoniae*. In this study, we could not find any association between CHR and country, intervention strategy, K-antigen type, or O-antigen type or colistin MIC value. For the ST, an association was found, however, only when C2 was used. In contrast, an association was found between CR and country, ST, O-antigen type, and K-antigen type. It is interesting to note that, based on an annual report from the European Centre for Disease Prevention and Control (ECDC) on antimicrobial consumption, Spain, which was found to have a higher number of CR isolates, also had a relatively higher consumption of polymyxins in hospitals compared to other European countries at least in the last period of the trial in which these isolates were collected (2016–2017) [26]. Also during 2017, colistin consumption in Spain remained relatively much higher compared to other countries included here [26]. Unfortunately, no data were available for a larger part of the duration of the trial (2013–2016).

For the ST, O-antigen, and K-antigen type, we often saw a relationship between the different molecular indicators and the STs which were found to have a statistically significant difference in the amount of CS and CR isolates, e.g., a specific K-antigen type was only present in combination with a specific ST and they were both found to have a higher proportion of CR isolates. For CHR, interestingly, no such pattern was found. CHR STs found to have an association with CR in this study are especially interesting since more than 90% of them are known to be associated with multidrug resistance [27,28,29]. In this study, MDR rates were high in CS, CHR, and CR isolates (>75%) and around one-third of the isolates determined to be CHR were CP-Kpn. CHR in MDR CP-Kpn is especially important to report as in most sites with a historically high proportion of CP-Kpn, colistin is empirically prescribed, and knowledge on the predilection towards development of CHR might facilitate the use of tailored antibiotic combinations instead.

In contrast to CS and CHR, the proportion of CR CP-Kpn isolates was relatively high (±30% vs. ±70%). Since CP-Kpn are more likely to be exposed to colistin, it is to be expected that CR rates will increase over time in this subset of isolates which is also reflected in this study with the far majority of CP-Kpn being CR. Interestingly, rates of CHR among CP-Kpn were similar to those of CS among CP-Kpn, and CHR rates also did not differ significantly between the baseline, CHX, SOD, and SDD. This also suggests that colistin exposure through SOD and SDD had no association with selection of isolates with a CHR phenotype.

For CR, we did find an association with CHX, SOD, and SDD. The association found between CR and CHX, SOD and SDD could, however, be linked to the relatively higher proportion of ST147 isolates, which was similarly found to be associated with CR compared to the baseline. Additionally, most ST147 isolates were originating from Spain. It was beyond the scope of this study to investigate the exact influence of each parameter on the increased proportion of CR isolates.

Finally, a closer look at the mechanism of colistin resistance for the resistant subpopulation of two CHR isolates showed that mutations in colistin resistance-conferring genes assessed in this study could not always be identified for each isolate. A recent study on CHR in wild-type *K. pneumoniae* isolates also reported that for 28% of mutants sequenced, no genetic modification was found in the panel of genes assessed [30]. Disruption of *mgrB* by insertion sequences was the most commonly found genetic modification in the resistant subpopulations. Additionally, a nonsense mutation and complete deletion of *mgrB* was found once and twice, respectively. A mutation outside of *mgrB* was only found once. The same study in wild-type *K. pneumoniae* isolates also reported a high number of *mgrB* genetic modifications (54%) [30]. A complete deletion was only found in 4% of the mutants while disruption by insertion sequences and other amino acid alterations were found in 28% and 22% of mutants, respectively [30]. Luo et al. also reported in their study that there was a high rate of *mgrB* insertional mutations and no mutations in *pmrAB* or *phoPQ* and stated that this was consistent with previous findings which showed that those genes had a significantly lower mutation rate compared to *mgrB*. However, they also stated that the high amount of *mgrB* disruptions may be related to the high prevalence of ST11 since this ST showed a significantly higher rate of *mgrB* disruptions compared to other STs in their study [31].

Though this study helps in expanding the knowledge on CHR in a clinical setting, there are also some limitations. Firstly, only limited sequencing data were available for CHR isolates. Future studies are needed to further assess the diversity of mechanisms of CHR and whether these mechanisms are ST-specific. Secondly, we cannot exclude confounding factors such as the usage of more colistin in some local settings, outbreaks with specific STs, and the prevalence of CP-Kpn which may vary between sites. Thirdly, we only studied the CS population for CHR. However, CHR can also exist as a (sub)proportion of CR isolates. Additionally, we only focused on CHR as a phenomenon in which there is a minor subpopulation with a MIC above the breakpoint in a major population with a MIC below the breakpoint. However, HR may also occur in entirely susceptible populations [32].

Given the large number of isolates screened, this study is a step forward in elucidating the prevalence and burden of CHR in common ST lineages of *K. pneumoniae*. Our data prompt for the development of more robust and simple diagnostics to enable implementation of HR detection on a larger scale, and for more structured studies to quantify the actual impact of CHR on treatment failures in patients receiving colistin.

## Figures and Tables

**Table 1:** Table contains a detailed breakdown of the PAP assay results (*n* = 288) including the reasons why isolates did not fulfil the definition of colistin heteroresistance (CHR) and the number of isolates per observed result. Of note, for some isolates that did not fulfil the definition of CHR but did show growth > 2 mg/L, there were multiple reasons why they were not classified as CHR. Sub-reasons are listed in order of importance. Each isolate was only included once, and if it fulfilled multiple sub-reasons, it was only included in the sub-reason considered most important. MIC = minimum inhibitory concentration, C1 = Classification 1, C2 = Classification 2.

Observed Results		No. of Isolates
Fulfilling C1	Growth on the plates containing 4 and/or 8 mg/L of colistin with a frequency of at least 1 × 10^−6^	25
Fulfilling only C2	Growth on at least all the plates containing colistin at a concentration up to and including eight-fold the MIC of the isolate at a frequency of minimally 1 × 10^−7^, minimum concentration at which there should be growth was 4 mg/L	83
Not fulfilling either classification but growth > 2 mg/L	Growth on 4 and/or 8 mg/L plate but frequency < 1 × 10^−7^	45
	For MIC 0.0625–0.5 mg/L: growth with frequency ≥ 1 × 10^−7^ on 8 mg/L plate but <1 × 10^−7^ on 4 mg/L plate	8
	For MIC 1–2 mg/L: growth with frequency ≥ 1 × 10^−7^ on 4 and/or 8 mg/L plate but <1 × 10^−7^ on plates ≥ eight-fold the MIC	30
	Growth with frequency ≥ 1 × 10^−7^ on plates ≥ eight-fold the MIC but frequency of 1 × 10^−7^ not reached on all plates < eight-fold the MIC	19
No growth at 4 and 8 mg/L		71
No growth at 1, 2, 4 and 8 mg/L		7

**Table 2:** Summary of mutations found in CR and CHR isolates. The table contains information on the mutations found in the resistant subpopulation of two confirmed CHR isolates (AN1505CP2 and IT0244CP) at different concentrations of the PAP assay plates on three separate assays. Additionally, the table contains information on three CR isolates with the same ST as IT0244CP (ST409). For AN1505CP2, there were no CR isolates with the same ST (ST323). CR = colistin-resistant, CHR = colistin-heteroresistant, PAP = population analysis profiling, MIC = minimum inhibitory concentration, ST = sequence type, IS = insertion sequence.

Isolate ID	MIC (mg/L)	ST	PAP Assay Plate Conc. (mg/L)	Mutations in *mgrB*	Other Mutations
IT0307CP (CR)	128	ST409		IS1R of IS1 family interruption at nt 107	
IT0636C (CR)	128	ST409		ISKpn34 of IS3 family interruption at nt 46	
IT0915C (CR)	64	ST409		IS903B of IS5 family interruption at nt 34	
IT0244CP (CHR 1st PAP)	0.5	ST409	2	ISKpn34 of IS3 family interruption at promoter	
			8	IS903B of IS5 family interruption at nt 117	
			16	IS1S of IS1 family interruption at promoter	
IT0244CP (CHR 3rd PAP)	0.5	ST409	2	IS1X2 of IS1 family interruption at nt 123	
AN1505CP2 (CHR 1st PAP)	1	ST323	4	Deleted	
			8	IS903B of IS5 family interruption at nt 70	
AN1505CP2 (CHR 2nd PAP)	1	ST323	8		*pmrB:* T157P
AN1505CP2 (CHR 3rd PAP)	1	ST323	8	Q30X	
			16	Deleted	

## Data Availability

All sequenced data generated and analyzed in this study were deposited at NCBI under Bioproject ID PRJNA948355.

## Supplementary Materials

The following supporting information can be downloaded at: https://www.mdpi.com/article/10.3390/antibiotics13030281/s1, Table S1: Summary of interventions including amount of isolates within each intervention per sample category. Table provides an overview of the contents of the different intervention strategies as well as the number of isolates sorted per sample category for both the complete collection (*n* = 676) and the selected subset (*n* = 288) of isolates. Surv = surveillance, PPS = point prevalence survey, Clin = clinical, CS = colistin sulphate, TBS = tobramycin sulphate, NYS = nystatin. ^1^ 2% mouthwash replaced by 1% oral gel after reports of oral mucosal adverse events. ^2^ Normally, regiment includes four days of IV cephalosporin, not included because of settings of moderate/high resistance. ^3^ Either sampling during 1 month wash-out/in period between intervention strategies or interruption of the intervention. Table S2: Overview of selected isolates. Table offers an overview of the selected isolates (*n* = 288) and their characteristics. BEL = Belgium, ESP = Spain, UK = United Kingdom, SVN = Slovenia, ITA = Italy, PRT = Portugal, Cat. = category, Surv = surveillance, PPS = point prevalence survey, Clin = clinical, CHX = chlorhexidine digluconate, SOD = selective oropharyngeal decontamination, SDD = selective digestive tract decontamination, ST = sequence type, K-antigen type = capsular polysaccharide antigen type, O-antigen type = lipopolysaccharide antigen type, Col MIC = colistin minimum inhibitory concentration, CHR = colistin-heteroresistant, MDR = multidrug-resistant, CP = carbapenemase producing, Undef. = undefined, ND = no data. Table S3: Number of MDR and CP-Kpn isolates. Tables gives summary of the number of MDR and CP-Kpn CS and CR isolates. For CS, MDR, and CP-Kpn, classification was not possible for five and three isolates, respectively. CS = colistin-susceptible, CR = colistin-resistant, MDR = multidrug-resistant, CP-Kpn = carbapenemase-producing *K. pneumoniae.* Table S4: Number of MDR and CP-Kpn isolates. Tables gives summary of the number of MDR and CP-Kpn selected CS and CHR isolates. For CS, MDR, and CP-Kpn, classification was not possible for four and three isolates, respectively, of which one isolate was also CHR following C2. For CHR, C1 + C2 represents the total amount of isolates fulfilling C2 whilst C2 alone represents isolates only fulfilling C2. CS = colistin-susceptible, CHR = colistin-heteroresistant, C1 = Classification 1, C2 = Classification 2, MDR = multidrug-resistant, CP-Kpn = carbapenemase-producing *K. pneumoniae.* Figure S1: Distribution of isolates across baseline and intervention strategies. Graphs show the number of isolates per strategy as well as the number of CR/CHR and CS/non-CHR isolates. Of note, C1 + C2 represents the total amount of isolates fulfilling C2 whilst C2 alone represents isolates only fulfilling C2. (A) CR per intervention strategy, (B) CHR per intervention strategy.
