# Supplementary material for: Assessment of Colistin Heteroresistance among Multidrug-Resistant Klebsiella pneumoniae Isolated from Intensive Care Patients in Europe
Source: Antibiotics (Basel). 2024 Mar 20;13(3):281. doi: 10.3390/antibiotics13030281 (PMC10967581; doi:10.3390/antibiotics13030281)
Supplement: Supplementary file 1 [file antibiotics-13-00281-s001.zip › Supplementary Figure S1.pdf]

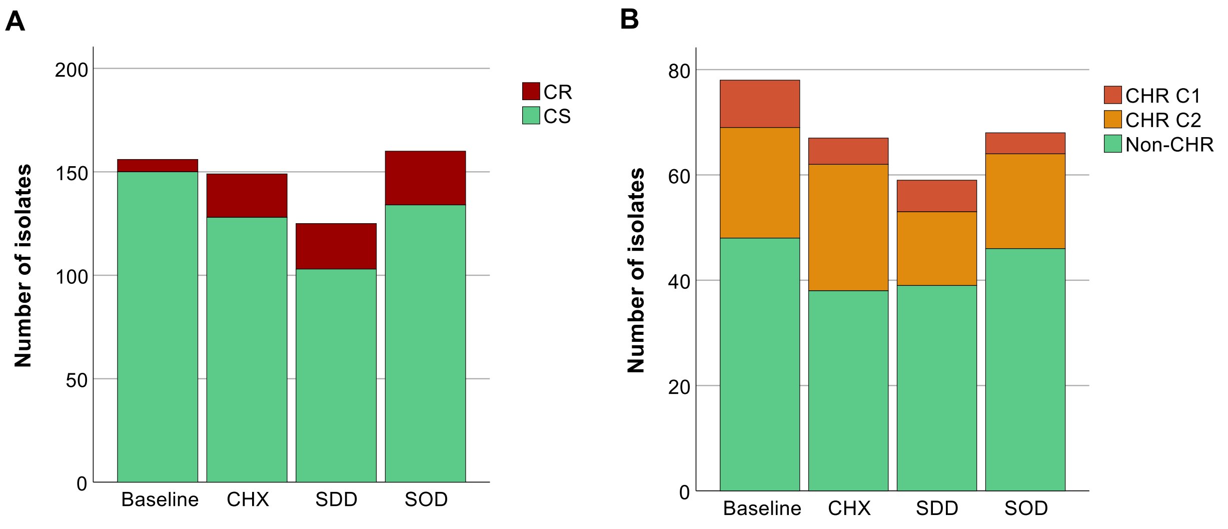

Figure S1: Distribution of isolates across baseline and intervention strategies. Graphs show the number of isolates per strategy as well as the number of colistin-resistant (CR)/colistin-heteroresistant (CHR) and colistin-susceptible (CS)/ non-CHR isolates. Of note, Classification 1 (C1) + Classification 2 (C2) represents the total amount of isolates fulfilling C2 whilst C2 alone represents isolates only fulfilling C2. (**A**) CR per intervention strategy, (**B**) CHR per intervention strategy. CHX = chlorhexidine digluconate, SOD = selective oropharyngeal decontamination, SDD = selective digestive tract decontamination.
