# Supplementary material for: Assessment of Colistin Heteroresistance among Multidrug-Resistant Klebsiella pneumoniae Isolated from Intensive Care Patients in Europe
Source: Antibiotics (Basel). 2024 Mar 20;13(3):281. doi: 10.3390/antibiotics13030281 (PMC10967581; doi:10.3390/antibiotics13030281)
Supplement: Supplementary file 1 [file antibiotics-13-00281-s001.zip › Supplementary Table S1.pdf]

Table S1: Summary of interventions including amount of isolates within each intervention per sample category. Table provides an overview of the contents of the different intervention strategies as well as the number of isolates sorted per sample category for both the complete collection (n = 676) and the selected subset (n = 288) of isolates. Surv = surveillance, PPS = point prevalence survey, Clin = clinical, CS = colistin sulphate, TBS = tobramycin sulphate, NYS = nystatin. [1]2% mouthwash replaced by 1% oral gel after reports of oral mucosal adverse events. [2]Normally regiment includes four days of IV cephalosporin, not included because of settings of moderate/high resistance. [3]Either sampling during 1 month wash-out/in period between intervention strategies or interruption of the intervention.

Intervention	Contents of intervention	No. of isolates			
			Surv	PPS	Clin
**Baseline**	Daily CHX 2% body washing + hand hygiene improvement program (WHO). Optional extra: CHX mouthwash (0.12 or 0.20%) if this was already part of regular care	All	106	35	15
		Selected	66	10	2
**Chlorhexidine digluconate (CHX)**	CHX 2% mouthwash/1% oral gel[1]	All	98	39	12
		Selected	52	13	2
**Selective oropharyngeal decontamination (SOD)**	Mouthpaste (oropharynx) containing: CS (0.19 million units), TBS (10 mg) and NYS (0.1 million units/0.5 g)	All	107	40	13
		Selected	49	14	5
**Selective digestive tract decontamination (SDD)[2]**	Mouthpaste (oropharynx): containing: CS (0.19 million units), TBS (10 mg) and NYS (0.1 million units/0.5 g) Suspension (gastrointestinal tract) containing: CS (1.9 million units), TBS (80 mg) and NYS (2.0 million units)	All	91	24	10
		Selected	46	11	2
**Wash-in/out period or interrupted**		All	63	14	9
		Selected	13	2	1
