# Supplementary material for: Assessment of Colistin Heteroresistance among Multidrug-Resistant Klebsiella pneumoniae Isolated from Intensive Care Patients in Europe
Source: Antibiotics (Basel). 2024 Mar 20;13(3):281. doi: 10.3390/antibiotics13030281 (PMC10967581; doi:10.3390/antibiotics13030281)
Supplement: Supplementary file 1 [file antibiotics-13-00281-s001.zip › Supplementary Table S2.pdf]

Table S2: Overview of selected isolates. Table offers an overview of the selected isolates (*n* = 288) and their characteristics. BEL = Belgium, ESP = Spain, UK = United Kingdom, SVN = Slovenia, ITA = Italy, PRT = Portugal, Cat. = category, Surv = surveillance, PPS = point prevalence survey, Clin = clinical, CHX = chlorhexidine digluconate, SOD = selective oropharyngeal decontamination, SDD = selective digestive tract decontamination, ST = sequence type, K-antigen type = capsular polysaccharide antigen type, O-antigen type = lipopolysaccharide antigen type, Col MIC = colistin minimum inhibitory concentration, CHR = colistin-heteroresistant, MDR = multidrug-resistant, CP = carbapenemase producing, Undef. = undefined, ND = no data.

Isolate ID	Country	Cat.	Intervention	ST	K-antigen type	O-antigen type	Col MIC (mg/L)	CHR	MDR	CP
**AN0157O**	BEL	Surv	Baseline	11	KL105	O2v2	0.25	2	+	+
**AN0222CP**	BEL	Surv	Baseline	530	KL54	O1/O2v2	0.5	-	-	-
**AN0328E**	BEL	Surv	Baseline	323	KL21	O3b	0.5	-	+	-
**AN0980CP**	BEL	Surv	Baseline	504	KL110	O1v1	0.5	2	+	-
**AN1092CP1**	BEL	Surv	Baseline	160	KL62	O1v1	0.25	-	+	-
**AN1505CP2**	BEL	Surv	SDD	323	KL21	O3b	1	1	+	-
**AN1876CP2**	BEL	PPS	SDD	2004	KL23	O2v2	1	-	+	-
**AN2029CP**	BEL	Surv	SDD	34	KL38	O3b	0.5	-	-	-
**AN2262CP**	BEL	PPS	SDD	219	KL114	O1/O2v1	1	-	+	-
**AN2496CP**	BEL	PPS	SDD	15	KL62	O1v1	0.25	2	+	-
**AN2784CP**	BEL	Surv	SDD	13	KL3	O1v2	0.5	2	+	-
**AN2982CP**	BEL	Surv	SDD	323	KL21	O3b	1	-	+	-
**AN2991E2**	BEL	Surv	SDD	3031	KL124	O3/O3a	0.5	-	-	-
**AN3090CP**	BEL	Surv	SDD	323	KL21	O3b	0.5	-	+	-
**AN3573CP**	BEL	Surv	CHX	45	KL24	O2v1	1	1	+	+
**AN3603CP**	BEL	Surv	CHX	147	KL64	O2v1	0.5	2	+	-
**AN4337CP2**	BEL	Surv	CHX	1709	KL70	OL102	0.25	-	-	-
**AN4440CP**	BEL	Surv	CHX	513	KL14	O3b	0.5	-	+	-
**AN5000CP**	BEL	PPS	SOD	39	KL149	O1v2	0.5	-	-	-
**AN5043CP**	BEL	Surv	Wash-in/out	13	KL3	O1v2	1	-	+	+
**AN5207CP**	BEL	PPS	SOD	307	KL102	O2v2	1	-	+	-
**AN5764E**	BEL	Surv	SOD	307	KL102	O2v2	0.25	-	+	-
**AN6344CP**	BEL	PPS	SOD	15	KL62	O1v1	0.125	2	+	-
**AN6484CP**	BEL	PPS	SOD	872	KL153	O2v2	0.25	-	-	-
**AN6502E**	BEL	Surv	SOD	884	KL150	O3b	1	-	+	-
**AN6511CP**	BEL	Surv	SOD	323	KL21	O3b	0.25	-	+	-
**AN6607CP**	BEL	Surv	Wash-in/out	1876	KL15	O4	0.25	-	-	-
**BC0043**	ESP	Surv	Baseline	147	KL64	O2v1	1	-	+	-
**BC0133CP**	ESP	PPS	Baseline	101	KL17	O1v1	0.25	-	+	+
**BC0275CP**	ESP	PPS	CHX	1083	KL132	O1v1	0.5	2	+	+
**BC0294E**	ESP	Surv	CHX	323	KL21	O3b	0.25	-	+	-
**BC0365**	ESP	Surv	CHX	392	KL27	O4	1	-	+	-
**BC0386**	ESP	PPS	CHX	485	KL22	O3b	1	-	+	-
**BC0495**	ESP	PPS	CHX	101	KL17	O1v1	0.0625	2	+	+
**BC0501**	ESP	PPS	CHX	20	KL28	O1v2	1	2	-	-
**BC0504**	ESP	Surv	CHX	101	KL17	O1v1	0.5	2	+	+
**BC0602**	ESP	Surv	SDD	405	KL151	O4	1	-	+	-
**BC0650**	ESP	Surv	SDD	17	KL25	O5	0.5	2	-	-
**CD0391E**	UK	Clin	Baseline	983	KL127	OL101	0.5	-	+	-
**CD0687E**	UK	Surv	Baseline	628	KL123	O3b	1	-	+	-
**CD0692E**	UK	Surv	SOD	37	KL7	OL104	0.5	-	ND	-
**CD0794C/E**	UK	Surv	SOD	340	KL123	O4	1	-	+	-
**CD0833E**	UK	Surv	SOD	20	KL28	O1v2	0.5	2	+	+
**CD0950CP**	UK	Surv	SOD	20	KL28	O1v2	1	-	+	+
**CD1069E**	UK	Surv	SOD	628	KL36	O3b	1	2	+	-
**CD1221CP**	UK	PPS	SOD	14	KL2	O1/O2v1	0.5	2	-	-

**CD1279O**	UK	Surv	SOD	678	KL15	O4	2	1	-	-
**CD1506E**	UK	Surv	CHX	45	KL24	O2v1	0.5	-	+	ND
**CD1695E**	UK	Surv	CHX	628	KL123	O3b	1	-	+	-
**CD1892E**	UK	PPS	CHX	15	KL19	O1/O2v2	1	1	+	-
**CD1901E**	UK	Surv	CHX	48	KL62	O1v1	1	2	+	-
**CD2009E**	UK	Surv	CHX	45	KL24	O2v1	0.5	-	ND	-
**CD2110E**	UK	PPS	SDD	307	KL102	O2v2	1	-	+	-
**CD2409E**	UK	Surv	SDD	628	KL123	O3b	0.5	2	+	-
**FE0004**	ESP	Surv	Baseline	3293	KL60	O3b	0.5	-	+	-
**FE0017**	ESP	Surv	Baseline	1427	KL67	OL101	0.25	-	+	-
**FE0022**	ESP	Surv	Baseline	1427	KL67	OL101	1	-	+	-
**FE0051**	ESP	Surv	Baseline	437	KL36	O4	0.5	-	+	-
**FE0117**	ESP	Surv	Baseline	391	KL30	O1v2	1	2	+	+
**FE0164**	ESP	PPS	Baseline	405	KL151	O4	1	1	+	-
**FE0245**	ESP	PPS	Baseline	76	KL21	O3/O3a	1	2	+	+
**FE0256**	ESP	Surv	Baseline	147	KL64	O2v1	0.5	-	+	+
**FE0389**	ESP	Surv	Baseline	11	KL13	OL104	0.5	-	+	-
**FE0467**	ESP	Surv	Baseline	437	KL36	O4	1	-	+	+
**FE0501**	ESP	Surv	Baseline	76	KL21	O3/O3a	1	2	+	+
**FE0710**	ESP	Surv	SDD	17	KL66	O2v2	2	2	+	+
**FE0777**	ESP	Surv	SDD	147	KL64	O2v1	2	-	+	-
**FE0780**	ESP	Surv	SDD	147	KL64	O2v1	1	-	+	-
**FE0820**	ESP	Surv	SDD	134	KL25	O5	1	2	+	+
**FE0883**	ESP	Surv	SDD	437	KL36	O4	0.5	-	+	+
**FE0992**	ESP	Clin	SDD	70	KL136	O1v2	0.5	2	+	-
**FE1068**	ESP	Surv	SDD	76	KL21	O3/O3a	0.125	-	-	-
**FE1279**	ESP	PPS	SOD	147	KL64	O2v1	0.25	-	+	-
**FE1312**	ESP	Surv	SOD	437	KL36	O4	0.5	2	+	-
**FE1385**	ESP	Surv	SOD	323	KL21	O3b	1	-	+	-
**FE1455**	ESP	Surv	SOD	76	KL21	O3/O3a	2	1	+	+
**FE1669**	ESP	Surv	SOD	101	KL17	O1v1	1	2	+	ND
**FE1762**	ESP	Surv	CHX	147	KL64	O2v1	0.5	-	+	+
**FE1764**	ESP	Surv	CHX	437	KL36	O4	0.5	2	+	+
**FE1825**	ESP	Surv	CHX	147	KL64	O2v1	1	-	+	+
**FE2000**	ESP	Surv	Wash-in/out	1117	KL117	O1v1	1	2	+	+
**GL0060E**	SVN	PPS	Baseline	14	KL105	O2v2	1	2	-	-
**GL0080E**	SVN	Surv	Baseline	15	KL2	O1v1	0.5	-	+	-
**GL0746E**	SVN	Surv	CHX	15	KL16	O1v1	0.5	2	+	-
**GL1380O**	SVN	PPS	Wash-in/out	11	KL24	O1v1	0.25	-	-	-
**GL1600E**	SVN	PPS	SDD	39	KL24	O1v1	0.5	-	+	-
**GL2607E**	SVN	PPS	SOD	15	KL112	O1v1	1	-	+	-
**GL2639E**	SVN	Surv	SOD	15	KL24	O1v1	1	-	+	-
**IT0032E**	ITA	Surv	Baseline	914	KL81	OL101	0.5	-	-	-
**IT0035E**	ITA	Surv	Baseline	15	KL24	O1v1	0.5	-	+	-
**IT0046CP1**	ITA	Surv	Baseline	307	KL102	O2v2	0.5	-	+	+
**IT0061O**	ITA	Surv	Baseline	15	KL24	O1v1	0.5	2	+	-
**IT0064CP**	ITA	Surv	Baseline	307	KL102	O2v2	1	-	+	+
**IT0067CP**	ITA	Surv	Baseline	307	KL102	O2v2	0.5	2	+	+
**IT0075CP**	ITA	Surv	Baseline	307	KL102	O2v2	2	-	+	+
**IT0080CP**	ITA	Surv	Baseline	307	KL102	O2v2	1	-	+	+
**IT0082CP3**	ITA	Surv	Baseline	307	KL102	O2v2	1	-	+	+
**IT0087CP**	ITA	Surv	Baseline	45	KL62	O2v1	0.5	-	+	+
**IT0097CP**	ITA	Surv	Baseline	307	KL102	O2v2	2	-	+	+
**IT0115CP**	ITA	Surv	Baseline	636	KL61	O5	0.5	1	+	+
**IT0132CP**	ITA	Surv	Baseline	101	KL17	O1v1	1	-	+	+
**IT0162CP**	ITA	Surv	Baseline	258/512	KL107	O2v2	0.25	2	+	+
**IT0164E**	ITA	Surv	Baseline	101	KL17	O1v1	0.5	2	+	-

**IT0195CP**	ITA	Surv	SDD	307	KL102	O2v2	1	-	+	+
**IT0218CP**	ITA	Surv	SDD	307	KL102	O2v2	2	-	+	+
**IT0238CP**	ITA	Surv	SDD	307	KL102	O2v2	0.25	-	+	+
**IT0244CP**	ITA	Surv	SDD	409	KL9	O3b	0.5	1	+	+
**IT0246CP**	ITA	Surv	SDD	258/512	KL107	O2v2	2	-	+	+
**IT0251CP**	ITA	Surv	SDD	307	KL102	O2v2	2	-	+	+
**IT0302CP**	ITA	Surv	SDD	409	KL9	O3b	0.25	2	+	+
**IT0329O**	ITA	Surv	SDD	409	KL9	O3b	0.125	2	+	+
**IT0341CP**	ITA	PPS	SDD	307	KL102	O2v2	2	-	+	+
**IT0349O**	ITA	PPS	SDD	23	KL1	O1v2	0.25	-	-	-
**IT0361E**	ITA	Surv	SDD	307	KL102	O2v2	0.125	1	+	+
**IT0370E**	ITA	PPS	SOD	409	KL9	O3b	1	-	+	-
**IT0374CP**	ITA	Surv	Wash-in/out	101	KL17	O1v1	0.25	-	+	+
**IT0385CP**	ITA	Surv	SOD	101	KL17	O1v1	0.25	-	+	+
**IT0386CP**	ITA	Surv	SOD	307	KL102	O2v2	1	-	+	+
**IT0394CP**	ITA	Surv	SOD	101	KL17	O1v1	0.5	2	+	+
**IT0403O**	ITA	Surv	SOD	307	KL102	O2v2	0.125	-	+	-
**IT0429**	ITA	Surv	SOD	101	KL17	O1v1	0.5	-	+	+
**IT0444CP**	ITA	Surv	SOD	307	KL102	O2v2	2	-	+	+
**IT0450CP2**	ITA	Surv	SOD	307	KL102	O2v2	0.5	-	+	+
**IT0481E**	ITA	Surv	SOD	985	KL39	O1v2	1	1	+	-
**IT0548E**	ITA	Surv	Interrupted	14	KL16	O1v1	1	-	+	-
**IT0591E**	ITA	Surv	Interrupted	107	KL103	O1/O2v1	0.5	2	+	-
**IT0598CP**	ITA	Surv	Interrupted	353	KL110	O3b	0.5	2	-	-
**IT0649O**	ITA	Surv	SOD	409	KL9	O3b	0.25	-	+	-
**IT0685O**	ITA	PPS	SOD	104	KL31	O3b	1	-	-	-
**IT0693O**	ITA	Surv	SOD	45	KL62	O2v1	0.25	-	+	+
**IT0700O**	ITA	Surv	SOD	409	KL9	O3b	2	-	+	-
**IT0728CP**	ITA	PPS	CHX	101	KL17	O1v1	0.25	2	+	+
**IT0733O**	ITA	Surv	Wash-in/out	15	KL24	O1v1	1	2	+	-
**IT0739E**	ITA	Surv	CHX	409	KL9	O3b	0.25	-	+	-
**IT0753CP**	ITA	Surv	CHX	258/512	KL107	O2v2	0.5	-	+	+
**IT0758CP**	ITA	Surv	CHX	101	KL17	O1v1	0.5	-	+	+
**IT0821**	ITA	Surv	CHX	258/512	KL107	O2v2	1	-	+	+
**IT0846O**	ITA	Clin	CHX	392	KL27	O4	0.25	2	+	-
**IT0849CP**	ITA	Surv	CHX	307	KL102	O2v2	1	-	+	+
**IT0866CP**	ITA	Surv	CHX	515	KL62	O1v1	0.125	-	+	+
**IT0867E**	ITA	Surv	CHX	515	KL62	O1v1	0.25	2	+	-
**IT0886E**	ITA	Surv	CHX	409	KL9	O3b	0.5	2	+	-
**IT0904E**	ITA	PPS	CHX	14	KL16	O1v1	1	-	ND	-
**IT0948E**	ITA	Surv	CHX	16	KL51	O3b	0.5	-	+	-
**IT0954O**	ITA	Surv	CHX	405	KL151	O4	1	-	+	-
**IT0966E**	ITA	Surv	CHX	16	KL51	O3b	0.25	2	+	-
**KG0086E**	BEL	Surv	Baseline	437	KL36	O4	1	2	+	-
**KG0275E**	BEL	Surv	Baseline	15	KL48	O1v1	0.125	-	+	-
**KG0312E**	BEL	Surv	SOD	437	KL36	O4	0.5	2	+	-
**KG0316E**	BEL	Surv	SOD	15	KL48	O1v1	0.125	1	+	-
**KG0641E**	BEL	Surv	SDD	15	KL48	O1v1	0.5	-	+	-
**KG0663E**	BEL	PPS	SDD	307	KL102	O2v2	0.5	2	+	-
**LB0060E2**	BEL	Surv	Baseline	37	KL118	OL101	0.5	-	+	-
**LB0063E**	BEL	Clin	Baseline	25	KL2	O1v2	0.5	2	-	-
**LB0066CP**	BEL	Surv	Baseline	258/512	KL107	O2v2	0.25	1	+	+
**LB0085E2**	BEL	Surv	Baseline	792	KL25	O3/O3a	0.5	2	-	-
**LB0323E**	BEL	Surv	Baseline	15	KL112	O1v1	1	2	+	-
**LB0343E**	BEL	PPS	Baseline	556	KL62	O2v1	1	1	-	-
**LB0464O**	BEL	Surv	Baseline	15	KL24	O1v1	0.5	2	+	-
**LB0500O**	BEL	Surv	Baseline	4398	KL126	OL101	1	-	-	-

**LB0743O**	BEL	Surv	Baseline	38	KL52	OL101	1	-	-	-
**LB0785E**	BEL	Surv	Baseline	405	KL111	O3/O3a	1	1	+	-
**LB1218CP**	BEL	Clin	CHX	258/512	KL107	O2v2	0.5	2	+	+
**LB1231E**	BEL	Surv	CHX	15	KL24	O1v1	0.5	2	+	-
**LB1326E**	BEL	Surv	CHX	11	KL105	O2v2	0.25	-	+	-
**LB1372E**	BEL	PPS	CHX	193	KL30	O1v2	0.25	1	-	-
**LB1577E**	BEL	Surv	CHX	Undef.	KL151	O4	1	2	+	-
**LB1695E**	BEL	Surv	CHX	45	KL24	O2v1	2	1	-	-
**LB1819E**	BEL	Surv	CHX	36	KL27	O2v2	0.125	2	+	-
**LB1945CP2**	BEL	Surv	CHX	258/512	KL107	O2v2	0.25	-	+	+
**LB1946CP**	BEL	Surv	CHX	258/512	KL107	O2v2	0.5	2	+	+
**LB2042E**	BEL	Surv	CHX	20	KL28	O1v2	1	-	-	-
**LB2337E**	BEL	Surv	CHX	348	KL62	O1v1	1	-	+	-
**LB2549E**	BEL	PPS	SOD	193	KL30	O1v2	0.5	2	+	-
**LB2648E3**	BEL	Clin	SOD	17	KL127	OL101	0.5	2	+	-
**LB2711E**	BEL	Surv	SOD	20	KL28	O1v2	2	-	+	-
**LB2716E**	BEL	Surv	SOD	11	KL15	O4	1	-	-	-
**LB2737E**	BEL	Surv	SOD	17	KL127	OL101	0.5	2	+	-
**LB2888O4**	BEL	Surv	SOD	405	KL151	O4	0.5	-	+	-
**LB3091E**	BEL	Surv	SOD	25	KL2	O1v2	0.5	2	+	-
**LB3189E3**	BEL	Surv	SOD	1214	KL51	O2v2	1	2	+	-
**LB3246E**	BEL	Surv	SOD	15	KL24	O1v1	1	-	+	-
**LB3311CP**	BEL	Surv	SOD	258/512	KL107	O2v2	1	-	+	+
**LB3880E**	BEL	Surv	Interrupted	405	KL151	O4	0.5	-	+	-
**LB4058CP**	BEL	Surv	Interrupted	894	KL58	O3b	0.125	-	+	+
**LB4191E**	BEL	Surv	Interrupted	48	KL124	O1v1	0.25	2	-	-
**LB4210E2**	BEL	Clin	Interrupted	405	KL151	O4	0.25	-	+	-
**LB4438E**	BEL	PPS	Interrupted	17	KL25	O5	0.5	1	-	-
**LB4884E**	BEL	PPS	SDD	628	KL123	O3b	1	-	+	-
**LB5023O**	BEL	Surv	SDD	147	KL64	O2v1	0.5	-	+	-
**LB5205CP**	BEL	Surv	SDD	405	KL151	OL102	1	-	+	+
**LB5245CP**	BEL	Surv	SDD	307	KL102	O2v2	0.5	1	+	+
**LB5310O**	BEL	Surv	SDD	15	KL24	O1v1	0.25	2	+	-
**LB5524E**	BEL	Surv	SDD	Undef.	KL151	O4	1	-	+	-
**LB5773E**	BEL	Surv	SDD	Undef.	KL151	O4	0.0625	-	+	-
**OT0108CP**	BEL	Surv	Baseline	258/512	KL106	O2v2	0.5	-	+	+
**OT0246E1**	BEL	Surv	Baseline	405	KL151	O4	0.5	-	+	-
**OT1141E**	BEL	Surv	SDD	405	KL151	O4	0.25	-	+	-
**OT1208E**	BEL	Surv	Wash-in/out	35	KL110	O1v1	0.5	-	+	-
**OT1486E**	BEL	Surv	CHX	3449	KL58	O5	2	2	+	-
**OT1558E**	BEL	PPS	CHX	35	KL110	O1v1	0.5	-	+	-
**PS0015E**	ESP	Surv	Baseline	326	KL25	O1v1	0.5	2	+	-
**PS0030E**	ESP	Surv	Baseline	11	KL13	O3b	0.5	-	+	-
**PS0161E**	ESP	Surv	Baseline	392	KL27	O4	1	-	+	-
**PS0202E**	ESP	Surv	Baseline	429	KL27	O4	0.25	-	+	-
**PS0230E**	ESP	Surv	Baseline	15	KL19	O1v1	0.125	-	+	-
**PS0231E**	ESP	PPS	Baseline	340	KL55	O3b	2	1	+	-
**PS0304CP**	ESP	Surv	Baseline	258/512	KL107	O2v2	0.5	2	+	+
**PS0477CP**	ESP	Surv	Baseline	258/512	KL107	O2v2	0.25	1	+	+
**PS0670E**	ESP	Surv	Baseline	35	KL22	O1v1	0.5	2	+	-
**PS0902E**	ESP	Surv	SOD	11	KL105	O2v2	1	-	+	-
**PS1641E**	ESP	PPS	SOD	15	KL19	O1v1	0.5	-	+	-
**PS1653E2**	ESP	PPS	SOD	34	KL117	O2v2	2	-	+	-
**PS1684E**	ESP	Surv	SOD	101	KL17	O1v1	0.5	-	-	-
**PS1694E**	ESP	Surv	SOD	392	KL27	O4	1	-	+	-
**PS1712E1**	ESP	Surv	SOD	1296	KL105	O3b	1	-	+	-
**PS1767E**	ESP	Surv	SOD	2604	KL60	O3/O3a	1	2	+	-

**PS1778E2**	ESP	PPS	SOD	11	KL60	O5	2	-	+	-
**PS1793E**	ESP	Surv	SOD	323	KL21	O3b	1	-	+	-
**PS1812E**	ESP	Surv	CHX	20	KL28	O1v2	1	-	-	-
**PS1901E**	ESP	Surv	CHX	307	KL102	O2v2	0.25	-	+	-
**PS1975E**	ESP	Surv	CHX	326	KL25	O1v1	2	-	+	-
**PS2052CP**	ESP	Surv	CHX	258/512	KL107	O2v2	0.25	-	+	+
**PS2061E**	ESP	PPS	CHX	2703	KL16	O3b	0.5	-	+	-
**PS2066CP**	ESP	Surv	CHX	15	KL112	O1v1	1	-	+	+
**PS2109E**	ESP	Surv	CHX	429	KL27	O4	2	-	+	-
**PS2249E1**	ESP	Surv	CHX	3451	KL105	O4	0.5	-	+	-
**PS2459E**	ESP	Surv	CHX	15	KL19	O1v2	1	2	+	-
**PS2892E**	ESP	PPS	SDD	556	KL62	O2v1	2	-	-	-
**PS2965E**	ESP	PPS	SDD	15	KL48	O1v1	1	-	+	-
**PS3264E1**	ESP	Surv	SDD	219	KL114	O1v1	0.125	-	+	-
**PS3273E1**	ESP	Surv	SDD	252	KL30	O1v1	1	1	+	-
**PS3274E**	ESP	Surv	SDD	326	KL25	O1v1	0.5	-	+	-
**PS3372E**	ESP	Surv	SDD	70	KL136	O1v2	1	1	+	-
**SA0044**	PRT	Surv	Baseline	15	KL19	O1v2	0.5	-	+	-
**SA0072**	PRT	Surv	Baseline	461	KL112	O1v2	0.5	-	+	-
**SA0165**	PRT	Surv	Baseline	15	KL19	O1v2	0.25	-	+	-
**SA0192**	PRT	Surv	Baseline	348	KL62	O1v1	0.5	-	+	-
**SA0215**	PRT	Surv	Baseline	13	KL3	O1v2	2	-	+	-
**SA0221**	PRT	Surv	Baseline	34	KL10	O1v2	0.25	-	+	-
**SA0241**	PRT	PPS	Baseline	Undef.	KL124	OL101	0.5	2	-	-
**SA0275**	PRT	Surv	Baseline	405	KL151	O4	0.5	-	+	-
**SA0287**	PRT	Surv	Baseline	348	KL62	O1v1	1	-	+	-
**SA0311**	PRT	Surv	Baseline	34	KL10	O2v2	1	1	+	-
**SA0449**	PRT	Surv	SDD	307	KL102	O2v2	2	-	+	-
**SA0465**	PRT	Surv	SDD	405	KL151	O4	1	-	+	-
**SA0478**	PRT	Surv	SDD	Undef.	KL64	O2v1	1	-	+	+
**SA0483**	PRT	Surv	SDD	15	KL19	O1v2	0.5	-	+	-
**SA0491**	PRT	Surv	SDD	15	KL19	O1v2	0.25	2	+	-
**SA0517**	PRT	PPS	CHX	45	KL24	O2v1	0.25	2	+	-
**SA0528**	PRT	Surv	CHX	15	KL19	O1v2	1	-	+	-
**SA0555**	PRT	PPS	CHX	348	KL62	O1v1	0.5	2	+	-
**SA0590**	PRT	Surv	CHX	11	KL25	O5	1	-	+	+
**SA0690**	PRT	Surv	SOD	348	KL62	O1v1	0.5	-	+	-
**SA0694**	PRT	Surv	SOD	1271	KL28	O1v2	1	-	-	-
**SA0741**	PRT	Surv	SOD	147	KL64	O2v1	0.5	-	+	+
**SA0746**	PRT	Surv	SOD	1695	KL163	O2v1	0.25	2	+	-
**UZ0571E**	BEL	PPS	Baseline	37	KL38	O3b	0.5	2	+	-
**UZ0911O**	BEL	Surv	Baseline	11	KL105	O2v2	1	2	-	-
**UZ1295O**	BEL	PPS	Baseline	15	KL62	O1v1	1	-	+	-
**UZ1812**	BEL	PPS	Baseline	1565	KL31	O3b	1	-	+	-
**UZ1963E**	BEL	Surv	Baseline	45	KL24	O2v1	2	1	-	-
**UZ2466E**	BEL	PPS	CHX	29	KL19	O1v2	0.25	-	+	ND
**UZ2482E**	BEL	Surv	CHX	307	KL102	O2v2	1	-	+	-
**UZ3622E**	BEL	Surv	Interrupted	1	KL45	O1v2	0.125	2	+	-
**UZ4152O**	BEL	PPS	SOD	37	KL23	O2v2	1	-	+	-
**UZ4205E**	BEL	Clin	SOD	152	KL149	O4	1	-	+	-
**UZ4233O1**	BEL	Surv	SOD	1427	KL155	OL101	1	-	-	-
**UZ4601CP/E**	BEL	Surv	SOD	1017	KL58	O3b	0.25	2	+	+
**UZ5772O**	BEL	Surv	SDD	17	KL25	O5	0.5	-	-	-
**UZ6482E**	BEL	Surv	SDD	Undef.	KL30	O1v2	1	-	-	-
**UZ6832E**	BEL	Clin	SDD	152	KL149	O4	0.25	2	+	-
**UZ6865O**	BEL	Surv	SDD	Undef.	KL30	O1v2	1	-	-	-
**VR0060E**	PRT	Surv	Baseline	307	KL102	O2v2	0.125	-	+	-

**VR0062E**	PRT	Surv	Baseline	45	KL24	O2v1	1	-	+	-
**VR0144E**	PRT	Surv	Baseline	307	KL19	O1v2	1	-	+	-
**VR0231E**	PRT	Surv	SDD	34	KL10	O1v1	1	2	+	-
**VR0321E**	PRT	Surv	SDD	4	KL128	O3b	0.0625	-	+	+
**VR0382CP**	PRT	Surv	SOD	922	KL64	O2v1	0.5	-	+	+
**VR0452E**	PRT	Surv	SOD	147	KL23	O2v2	0.5	2	+	-
**VR0533E**	PRT	Clin	SOD	280	KL64	O2v1	1	-	+	-
**VR0556E**	PRT	Clin	SOD	377	KL15	O4	1	-	+	-
**VR0588E/C**	PRT	Clin	SOD	34	KL64	O2v1	0.25	2	+	+
**VR0601E**	PRT	Surv	CHX	147	KL1	O1v2	0.5	-	+	+
**VR0664E**	PRT	Surv	CHX	15	KL10	O2v2	0.25	1	+	+
**VR0750E**	PRT	Surv	CHX	34	KL25	O5	0.25	2	+	-
**VR0774E**	PRT	Surv	CHX	11	KL17	O1v1	2	-	+	+
**VR0782E**	PRT	Surv	CHX	870	KL102	O2v2	0.5	2	+	+
