# Supplementary material for: Assessment of Colistin Heteroresistance among Multidrug-Resistant Klebsiella pneumoniae Isolated from Intensive Care Patients in Europe
Source: Antibiotics (Basel). 2024 Mar 20;13(3):281. doi: 10.3390/antibiotics13030281 (PMC10967581; doi:10.3390/antibiotics13030281)
Supplement: Supplementary file 1 [file antibiotics-13-00281-s001.zip › Supplementary Table S3.pdf]

Table S3: Number of MDR and CP-Kpn isolates. Tables gives summary of the number of MDR and CP-Kpn CS and CR isolates. For CS, MDR, and CP-Kpn, classification was not possible for five and three isolates, respectively. CS = colistin-susceptible, CR = colistin-resistant, MDR = multidrug-resistant, CP-Kpn = carbapenemase-producing *K. pneumoniae.*

	Total	MDR		CP-Kpn	
		+	-	+	-
**CS**	597	539	53	174	420
**CR**	79	78	1	57	22
