# Supplementary material for: Assessment of Colistin Heteroresistance among Multidrug-Resistant Klebsiella pneumoniae Isolated from Intensive Care Patients in Europe
Source: Antibiotics (Basel). 2024 Mar 20;13(3):281. doi: 10.3390/antibiotics13030281 (PMC10967581; doi:10.3390/antibiotics13030281)
Supplement: Supplementary file 1 [file antibiotics-13-00281-s001.zip › Supplementary Table S4.pdf]

Table S4: Number of MDR and CP-Kpn isolates. Tables gives summary of the number of MDR and CP-Kpn selected CS and CHR isolates. For CS, MDR, and CP-Kpn, classification was not possible for four and three isolates, respectively, of which one isolate was also CHR following C2. For CHR, C1+C2 represents the total amount of isolates fulfilling C2 whilst C2 alone represents isolates only fulfilling C2. CS = colistin-susceptible, CHR = colistin-heteroresistant, C1 = Classification 1, C2 = Classification 2, MDR = multidrug-resistant, CP-Kpn = carbapenemase-producing *K. pneumoniae.*

	Total	MDR		CP-Kpn	
		+	-	+	-
**CS (selected)**	288	244	40	80	205
**CHR C1**	25	19	6	9	16
**CHR C2**	83	72	10	23	59
